# Evaluating the effects, implementation experience and political economy of primary healthcare facility autonomy reforms within counties in Kenya: a mixed methods study protocol

**DOI:** 10.1136/bmjph-2024-001156

**Published:** 2024-10-15

**Authors:** Anita Musiega, Beatrice Amboko, Beryl Maritim, Jacinta Nzinga, Benjamin Tsofa, Peter Mwangi Mugo, Ethan Wong, Caitlin Mazzilli, Wangari Ng'ang'a, Brittany L Hagedorn, Gillian Turner, Anne Musuva, Felix Murira, Nirmala Ravishankar, Edwine Barasa

**Affiliations:** 1 Health Economics Research Unit, KEMRI-Wellcome Trust Research Programme, Nairobi, Kenya; 2 Health Systems and Research Ethics, KEMRI-Wellcome Trust Research Programme, Kilifi, Kenya; 3 Bill & Melinda Gates Foundation, Seattle, Washington, USA; 4 ThinkWell, Washington, DC, USA; 5 ThinkWell, Nairobi, Kenya; 6 Health Economics Research Unit, KEMRI-Wellcome Trust Research Programme, Kilifi, Kenya; 7 Nuffield Department of Medicine, Center for Tropical Medicine and Global Health, University of Oxford, Oxford, UK

## Abstract

**Introduction:**

There is a growing emphasis on improving primary health care services and granting frontline service providers more decision-making autonomy. In October 2023, Kenya enacted legislation mandating nationwide facility autonomy. There is limited understanding of the effects of health facility autonomy on primary health care (PHC) facilities performance. It is recognized that stakeholder interests influence reforms, and gender plays a critical role in access to health and its outcomes. This protocol outlines the methods for a study that plans to evaluate the effects, implementation experience, political economy, and gendered effects of health facility autonomy reforms in Kenya.

**Methods and Analysis:**

The research will use a before-and-after quasi-experimental study design to measure the effects of the reform on service readiness and service utilization, and a cross-sectional qualitative study to explore the implementation experience, political economy, and gendered effects of these reforms. Data to measure the effects of autonomy will be collected from a sample of 80 health facilities and 1600 clients per study arm. Qualitative interviews will involve approximately 83 facility managers and policymakers at the county level, distributed across intervening (36), and planning to intervene (36) counties. Additionally, 11 interviews will be conducted at the national level with representatives from the Ministry of Health, the National Treasury, the Controller of Budget, the Council of Governors, the Auditor General, and development partners. Given the uncertainty surrounding the implementation of the reforms, this study proposes two secondary designs in the event our primary design is not feasible – a cross-sectional study, and a quasi-experimental interrupted time series design. The study will use a difference-in-difference analysis for the quantitative component to evaluate the effects of the reforms, while using thematic analysis for the qualitative component to evaluate the political economy and the implementation experience of the reforms.

**Ethics and Dissemination:**

*This study was approved by the Kenya Medical Research Institute Scientific and Ethics Review Unit (*KEMRI/SERU/CGMR-C/294/4708*) and the National Commission for Science, Technology and Innovation (*NACOSTI/P/23/28111*). We plan to disseminate the findings through publications, policy briefs and dissemination workshops*

## Background

Primary Healthcare (PHC) is an entire society’s approach to delivering timely, comprehensive, and people-centered health services as close as possible to people’s everyday surroundings [1]. PHC prioritizes the delivery of essential health promotion, and disease prevention, in addition to treatment, rehabilitation, and palliative care, and is a crucial element in Universal Health Coverage (UHC) as it ensures, equitable, and efficient access to healthcare that prioritizes the needs of individuals [2]. First referral hospitals and peripheral outpatient health facilities are integral in delivering facility based PHC services in low and middle-income countries (LMICs). Evidence shows that how health facilities are organized and managed determines their effectiveness, and the extent to which they meet service delivery goals [1,3].

Health facility autonomy is a crucial aspect of the organizational structure and management of health facilities. Health facility autonomy refers to the level of control and influence that health facilities have over key functions health facility administration, financial management, procurement, human resource management and strategic management [4]. Historically, health facility autonomy reforms emerged as part of new public management prescriptions for hospital reforms, and entailed both decentralization of functions to hospitals, and exposure to the market [5]The level of autonomy granted to public facilities has been shown to directly effects their performance [2]. Unfortunately, in low- and middle-income countries, most public primary healthcare facilities lack autonomy over most of the managerial functions. For instance, less than 40% of facilities in low- and middle-income countries have autonomy to retain and spend their revenue [2].

Historically, health facility autonomy reforms have been introduced to enhance the efficiency of health facility operations and reduce their reliance on government budgets. Autonomy reforms have also been part of broader decentralization reforms aimed at improved efficiency and equity of health systems as well as enhance local participation and accountability [4,6–8].

However, like broader decentralization reforms, there is mixed evidence regarding whether granting primary healthcare facilities autonomy improves health facility performance in LMICs. For example, a study conducted in India found no significant relationship between health facility decision-making autonomy and performance [9]. In Uganda, a study that explored the health facility decision space found increased autonomy to be positively associated with the availability of drugs but negatively associated with quality of care and performance management [10]. In Tanzania, increased autonomy has had varying levels of effects on performance, linked to the functionality of the facility governance committees [11]. The mixed evidence may be attributed to context-specific factors influencing the effects on autonomy [12]. These mixed findings notwithstanding, there is increasing attention to leveraging health facility autonomy to improve the performance of health facilities and to enhance the role of the people in health service delivery, more so in PHC facilities. In addition, often health system reforms are experienced differently across genders, it is important that policy reforms consider the gendered impact to mitigate for unintended consequences.

Methodologically, most studies that have documented the effects of autonomy reforms have been qualitative [4,7,8,13] and the few quantitative studies in this area are cross sectional presenting attribution challenges [9]. This study proposes to adopt a mixed methods approach, incorporating a qualitative inquiry, and a range of quantitative methods (quasi experimental before and after, cross-sectional, and interrupted time series designs) aimed at estimating the effect of autonomy reforms. The study also proposes to conduct a political economy, and gender analysis of autonomy reforms, both of which have not been examined in previous studies of autonomy.

### Autonomy reforms in Kenya

Kenya has been on a path to improve service delivery through devolution and more recently, increased investments in primary healthcare services [14]. Kenya devolved health service delivery to county governments in 2013. Health facilities enjoyed some level of autonomy (health facility administration, financial management, procurement, human resource management and strategic management), pre-devolution, through the health management services fund (HMSF) for hospitals and Health Sector Services Fund (HSSF) for health centers and dispensaries which was shown to be effective in enhancing service delivery [13]. However, following the devolution of health services in 2013, health service delivery was reorganized with county health management teams (CHMTs) taking up the responsibility for the management of health services, and usurping the autonomy of sub-county and facility management committees [7].

Overtime, various counties have since acknowledged the need for facility managerial autonomy and have allowed facilities some level of financial and procurement autonomy [15]. Across these counties, this has been done through various means including enactment of county level legislation, and/or adoption of county level policies to facilitate health facility autonomy. However, many counties have continued to limit the autonomy of health facilities. At the time of writing this protocol, the country had recently enacted a new Facility Improvement Funds (FIF) law that is aimed at according some level of financial and procurement autonomy to public sector health facilities across the country [16].The law requires that facilities in the country are allowed to retain and spend their own source revenue. This law mandates all counties to implement autonomy reforms. However, the government was yet to establish guidelines for the implementation of this new law. Furthermore, due to the devolution of the management of health services in the country, the adoption and implementation of this new law is likely to depend on individual county leadership, unlike before the devolution when national laws were adopted exactly as guided by the national government. This will likely lead to variations in uptake of facility autonomy across counties in terms of both time of implementation and mechanism of implementation. These variations in implementation provides an opportunity to evaluate the effectiveness, and implementation experience of autonomy reforms.

In addition, it is crucial to consider the gendered effect of these autonomy reforms. Within Kenya, and in other African countries, there is a gendered influence in health leadership progression, with women often lagging [17,18]. In Kenya, it has also been shown that policies that are gender sensitive have had a positive influence on women in leadership [17]. It is important to assess whether the autonomy reforms will reflect the representation of women in decision making. In addition, gender dynamics often play a significant role in healthcare utilization, access, and outcomes [19,20]. This study will explore whether the autonomy reforms have differential effects on men and women, considering factors such as health system leadership positions, access to services, health outcomes, and the involvement of women in decision-making processes at the community and facility levels. Addressing gender-specific challenges and opportunities within the framework of PHC and facility autonomy will contribute to a more comprehensive and inclusive understanding of the healthcare system in Kenya.

It is important to document these autonomy reforms, their successes, and failures, to provide learning points for counties willing to implement similar reforms in the future, and for other LMICs keen on enhancing PHC provider autonomy. This study seeks to examine the effects, implementation experience, political economy and gendered effect of facility autonomy reforms among counties in Kenya.

## Methods

To address the above objectives, this study will adopt a concurrent mixed-method study design. The quantitative component of the study will entail a before and after quasi-experimental study as the primary design. The qualitative study will entail 1) a process evaluation and 2) a political economy analysis of the facility autonomy reforms. Both the quantitative and qualitative elements of the study will incorporate a gender lens. This study will be carried out between January 2023 and February 2026.

### Evaluation Framework - Theory of change

**Figure 1:**
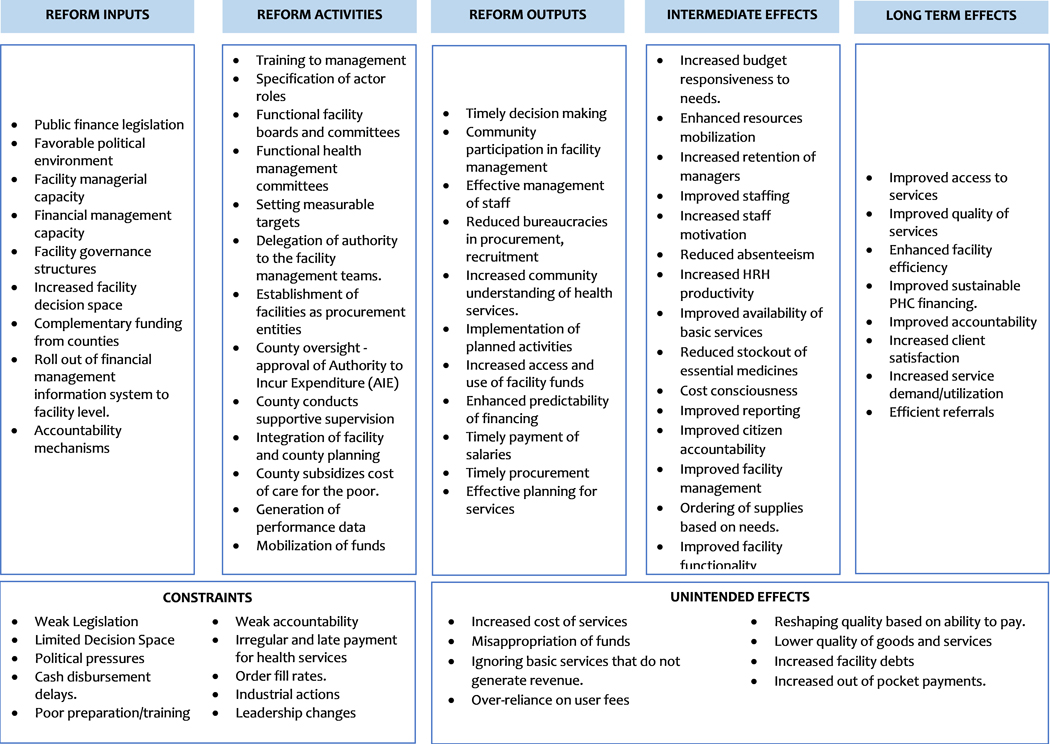
Theory of Change

This study will be guided by a Theory of Change (ToC) (Figure 1) which we developed from reviewing literature on the relationship between facility autonomy and facility performance, and a co-creation workshop that brought together 25 health sector stakeholders drawn from the national and county level, and government and development partners. The ToC assumes that facility autonomy spans key functions of the facility including strategic management, financial, procurement, human resource management, and facility administration [7]. This autonomy will require effective legislation, a favorable political environment, facility managerial capacity, facility capacity to claim reimbursements, facility governance structures, increased facility decision space, complementary funding from counties, roll out of financial management information system to facility level, and established accountability mechanisms [4]. The key activities will include training to management, specification of actor roles, functional facility boards and committees, functional health management committees, setting measurable targets, delegation of authority to the facility management teams, establishment of facilities as procurement entities, county oversight - approval of Authority to Incur Expenditure (AIE), supportive supervision by the county and sub-county health management teams, integration of facility and county planning, subsidization of the cost of care for the poor to guard against inequity, generation of performance data and mobilization of funds [4,7,21–23].

The immediate outputs expected from the inputs and activities include, timely decision-making, enhanced community participation in facility [7,24,25], effective management of staff [24,25], reduced bureaucracies in procurement and recruitment, increased community awareness of services [7] implementation of planned activities [7,26] increased access and use of facility funds [7,26], enhanced predictability on financing [4], timely payment of salaries [4,26], timely procurement, [4,26], and finally, effective planning for services.

The expected medium-term outcomes include increased budget responsiveness to needs, enhanced resource mobilization, increased retention of managers, improved staffing, increased staff motivation [7,26,27], reduced absenteeism, increased HRH productivity [4,24,25], improved availability of basic services, reduced stockout of essential medicines, cost consciousness, improved reporting, improved citizen accountability and participation, improved facility management and ordering of medicines and supplies based on need. These medium-term goals should then result in the achievement of the following long-term goals: improved access to services, improved quality of services, enhanced facility efficiency, improved sustainable PHC financing, improved accountability, Increased client satisfaction, increased service demand/utilization and efficient referrals [4,7,26,27].

From the literature, the effect of autonomy reforms is subject to context which is determined by institutional, socio-economic, and geographic factors [12]. As such, we will monitor constraints that are likely to influence the effectiveness of the reform including weak legislation, dysfunctional boards/committees, limited decision space, political pressures, cash disbursement delays, and poor preparation/training [4,28].

Finally, autonomy reforms may have unintended negative consequences including increased cost of services, misappropriation of funds, ignoring basic services that do not generate revenue, over-reliance on user fees, reshaping quality based on ability to pay, lower quality of goods and services, increased facility debts and increased out of pocket payments. We will monitor the possible unintended consequences and document the efforts by counties to mitigate them.

### Evaluating the effects of autonomy reforms

#### Primary study design

We will employ a before and after quasi-experimental study to measure the effects of health facility autonomy on health facility readiness, functionality, service utilization and client satisfaction of health facility autonomy reforms. We will conduct an assessment pre-implementation of the FIF reforms and a post-assessment following 12 months of implementation of FIF reforms. The primary outcomes of interest will be health facility readiness.

#### Secondary study design

There are uncertainties about the roll-out of autonomy, with possibilities that might compromise the proposed study design – (quasi experimental before and after design). The pace of autonomy reforms roll-out could be slow, with the implication that the selected intervention counties do not convert to intervention counties (i.e., autonomy reforms are not implemented in these counties over the 12-month study period). To mitigate this, we will sample 4 additional intervention counties that have already implemented autonomy reforms. We will use a cross-sectional study design comparing these 4 counties that are already implementing autonomy, with the counties that do not have autonomy (control counties). This design will also ensure that we can measure effects in the event the time of the study is not sufficient to measure effects within counties that are just rolling out autonomy.

Further, we will use an Interrupted Time Series (ITS) design to evaluate the effects of the reforms in the counties that will have rolled out the reforms across the country. We will collect and analyze monthly DHIS data on key performance indicators from primary health facilities across selected counties in Kenya, spanning 24 months before the introduction of autonomy reforms in a county, and 24 months after its introduction. Exact times for pre-intervention and post-intervention phases will vary based on the exact time that autonomy reforms were introduced in a county.

#### Study Population

We intend to assess selected counties, selected facilities, and clients receiving services from the selected facilities. For the county assessment, the study population will be the 47 counties from which we have selected 12 counties −4 implementing autonomy reforms, 4 planning to implement, and 4 controls. For the facility assessment, the study population will be all county owned public facilities within the selected counties across all levels of service provision (Table 1). For the client exit interviews the study population will be all patients or carers’ whose patients will have received services from the selected facilities on the day and time of the survey. These will include patients’ or carers’ seeking services such as outpatient services, antenatal services, postnatal services, child welfare services, management of chronic illness, maternity services, and those discharged from inpatient care.

#### Sample size and sampling

There will be three levels of sampling for this study. The first level is county sampling, where we will purposively sample 4 counties already implementing autonomy reforms, 4 planning to implement the intervention, 4 not implementing as control. From the sampled counties, we will randomly sample 80 health facilities per group, assuming a mean difference of 10% in facility readiness between the two groups and a population variance of 400 across the groups using 80% power, 95% confidence level and a design effect of 1.2. Because the autonomy reforms have the biggest effects on facilities that raise revenue, we will sample all level 4, 5 and 6 facilities from the selected counties. For the remaining facilities, we will then randomly sample level 2s and 3s. The numbers will be proportionate, targeting a maximum 20 facilities per county. For clients we will randomly sample a minimum of 1600 clients exiting the health facilities per group, assuming a mean difference of 10% in client satisfaction between the two groups and a population variance of 55 across the groups using 80% power, 95% confidence level, and a design effect of 3. To calculate the variance, we took an average of the outcome of the interest in the control and intervention (expected) [29–31]. We divide the 1600 equally among the selected facilities to achieve a minimum sample of 20 per facility as is recommended for cluster randomized trials [29,31]. The following sample size calculation formula was used:

$$n=(Z_{\alpha /2}+Z_{\beta })sqrd*2*\sigma^{2}/d^{2}$$

Where:

Zα/2 =1.96 (5% significance)

Zβ = 0.84 (80% power)

σ2 = population variance of 59 (standard deviation of 19 per group), and

d = mean difference of 8% between the two groups

### Data Collection

We will use trained research assistants to collect data using structured questionnaires on a web-based electronic platform. These questionnaires will be administered in the period preceding, and 12 months after the rollout of autonomy reforms. The health facility questionnaire will be administered to the health facility in charge and other health workers with information on the various sections. It will collect data on the facility profile, target population, services provided, availability of medicine, availability of laboratory services, availability of basic equipment, and service utilization (Supplemental Table 1). The health facility assessment questionnaire will evaluate the impact of autonomy on service availability, access to basic equipment, availability of medicines, service utilization, and facility financing (see Supplemental Table 1). The client exit interview tool will measure the effects of health facility autonomy on some subjective and objective indicators around service availability, and client satisfaction. The exit interviews will seek to establish whether health facility autonomy influences the availability of services and client satisfaction. It will entail questions about the services sought, whether the clients received the services, the client’s perspective on the cost of services, the client’s perspective on facility cleanliness and health worker attitude, how often the clients visit the health facility, and whether clients are satisfied with the services (Supplemental Table 1)

### Data analysis

We will compute means, medians, and standard deviations of all study variables and key sample characteristics at the baseline and end line. To determine the effects of autonomy reforms on service utilization and client satisfaction, we will use the quasi-experimental Difference in Difference (DiD) analysis. We will first compute the differences in means between the baseline and end line for each study group, then the differences in mean between the intervention and control.

Thereafter, we will estimate the effect using multivariate regression analysis. This means that we will calculate the DiD while controlling for other covariates that are likely to affect outcomes such as facility average insurance coverage, average distance to facility for facility catchment population, and health worker density, facility size, and insurance coverage. The unit of analysis will be the health facility and the outcomes of interest will be service utilization and client satisfaction.

### Qualitative study

#### Study Design

The qualitative aspects of the study will address objectives 2, 3, and 4 of the study. We will employ a cross-sectional qualitative study design to 1) assess the gendered effect of facility autonomy reforms 2) assess fidelity and implementation experience of hospital autonomy reforms and 3) examine the political and economic factors that influence hospital autonomy reforms.

##### Gendered Analysis

The gender analysis will entail examining the reform for the gendered effects. We propose to use the 4R method to conduct the gendered analysis – Responsibility, in terms of the gendered allocation of responsibility, the gendered allocation of resources, the reason behind the gendered allocations and the realization – what would improved gender equality look like [32].

##### Process Evaluation

The process evaluation of autonomy reforms will entail examining the emergence of autonomy reforms, the fidelity of the implementation reforms, the implementation processes, and the reasons for the resultant outputs or outcomes. In examining the emergence of the autonomy reforms, we examine the factors that led to the reforms and the adopted approaches. In examining the implementation fidelity, we will seek to compare the reforms as outlined in the FIF guidelines and laws (dejure policies) and what is implemented “on the ground” (defacto policies). In examining the processes, we will seek to document actors’ experiences, the implementation process, adaptations triggered by the reforms, unintended consequences, and how contextual issues shaped the reforms. In examining the resultant outputs or outcomes, we will present the results of the quantitative aspects of the study and seek to enrich the findings with qualitative data. We will examine implementation fidelity based on what is articulated in the implementation plans of health facility autonomy reforms in the intervention counties.

##### Political Economy Analysis

We will conduct a problem-driven political economy analysis (PEA). This will entail the exploration of actor interests and incentives, the dejure and defacto policies, and how they interact to influence health facility autonomy [33]. We propose to use a modified World Bank problem-driven PEA [34]. We adopt this approach because, from the literature, the success of autonomy reforms is linked to the institutions, actors, historical conditions, and power dynamics [4,7,15,26]. The problem-driven political economy analysis has three aspects: i) description of the problem ii) Institutional analysis which entails mapping out all the government organizations involved in (financial) autonomy, and the legal and policy documents that relate to the problem, and finally, we will iii) identify political economy drivers – specifically this will entail describing why things exist the way they do[35]. In exploring the drivers, we will consider three structures of drivers – structural (historical legacies, status of poverty, rent/rent distributions), institutional, and actors/stakeholders[35]. To assess the stakeholders, we propose to adopt Sparkes’ classification of actors: interest group politics, bureaucratic politics, budget politics, leadership politics, beneficiary politics, and external politics [33].

#### Study population

The study population will include both national and county-level stakeholders involved in health facility autonomy reforms. We will include national, county, and facility stakeholders involved in health financing and PHC service delivery decision-making. These will include stakeholders from health departments, finance departments, and county assembly, who were involved in the health financing reforms.

#### Sample size and sampling

The main data collection method will be in-depth interviews and document reviews. For the in-depth interview, we anticipate interviewing about 83 participants; however, this will be guided by the need to have county balance as the reforms are county specific, and saturation of data. These respondents will be purposively selected based on their knowledge and involvement in health facility autonomy reforms. Based on the number of people holding these positions, we anticipate conducting a total of 11 interviews at the national level and 12 interviews each from 3 already intervening counties, 3 planning to intervene counties (Table 2). For the document reviews, we will review documents related to health facility autonomy including the PFM laws, budget documents, facility reports, minutes, and county bills.

#### Data Collection

We will collect data using in-depth interviews and document reviews. In-depth interviews will target national and county-level officials involved in facility autonomy reforms (Table 2). We will collect relevant data from documents that relate to facility autonomy reforms.

#### Qualitative Data Analysis

The audio recordings will be converted into text format using Microsoft Word and then processed in NVIVO software for coding. Analysis of the data will follow a thematic approach. Thematic analysis is a method that guides the identification, organization, description, analysis, and reporting of themes founds in a data set [36]. The data analysis will follow six steps: familiarizing with the data, generating initial codes, searching for themes, reviewing themes, naming themes, and writing the report [36,37]. We will report the gendered effects of the reforms, implementation process and political economy as detailed in the list of analysis areas and outcomes.

### Data Management

#### Methodological rigor

To ensure the robustness of our research, we will implement various measures. First, we will conduct a literature review and a co-creation workshop to validate the theory of change that will guide the development of indicators. Second, we will document our entire research process and provide clear rationales for our study choices. Third, we will align our analysis with the theory of change and using both quantitative and qualitative designs for triangulation. Fourth, we will extensively train our data collectors, and conduct pilot studies in Kisumu, Mombasa, Makueni, and Kiambu counties. Fifth, when developing tools, we will adopt measures from established instruments, control for extraneous variables use a large randomly selected sample. Methodological rigor in qualitative aspects will involve peer debriefing, reflexivity, and respondent validation during a dissemination workshop, while a pilot study will test and refine our research protocols before the main study. Adjustments based on pilot feedback will contribute to our overall methodological robustness in future research endeavors.

We will ensure data safety; no identifiable information will be stored with survey responses. Survey responses will be maintained on secure, password-protected servers at KEMRI-Wellcome Trust Research Programme (KWTRP). Deidentified data files will be shared with the research team via secure file transfer and will be maintained on password-protected devices.

We will develop a database capturing data from the questionnaires in REDCap or equivalent software in collaboration with the KEMRI-Wellcome Nairobi data team. We will check the data for errors and import it into R for analysis. We will collate data from these questionnaires on a central server maintained by the KWTRP after being de-identified and encrypted for transmission over the internet. De-identified data shared with KWTRP will be stored in secure servers with password-protected access and as guided by KEMRI – Wellcome Trust ICT and Data Management policies. Data held on these servers are backed-up in similar password-protected mirror servers at another location within the KEMRI – Wellcome Trust.

#### Ethical Considerations

This study has received ethics approval from the Scientific and Ethics Review Unit (SERU) at Kenya Medical Research Institute (KEMRI) approval number KEMRI/SERU/CGMR-C/294/4708. The study has also received approval from the National Commission for Science, Technology and Innovation (NACOSTI) approval number NACOSTI/P/23/28111. Recognizing potential participant anxiety related to autonomy within counties, we will conduct low-risk interviews in private locations to ensure confidentiality. All study data, including transcripts and documents, will be deidentified and securely stored. Informed consent will be provided to all participants, with participants receiving comprehensive information sheets and consent forms in either English or Swahili based on their preferences.

#### Communication and Dissemination of results

Throughout the study period, we plan to conduct dissemination workshops with policymakers. Initially, we organized a co-creation workshop during the protocol development phase to validate the theory of change. We will host a dissemination workshop following the preliminary analysis of baseline data, and another after the final data collection and analysis. Our study is integrated within the Ministry of Health and the Council of Governors, who are key collaborators. Additionally, we intend to publish various aspects of the study and present our findings at conferences.

## Supplementary Material

Supp1

## Figures and Tables

**Table 1:** Selected Counties with distribution of facilities

Group	County	Level 2	Level 3	Level 4	Level 5	Level 6	Total Facilities in county
Controls	Tana River	44	5	3	0	0	52
	Migori	115	19	14	0	0	148
	Embu	88	9	4	1	0	102
	Kirinyaga	47	22	4	0	0	73
Already Implementing	Makueni	189	39	12	0	0	240
	Mombasa	40	8	4	1	0	53
	Nakuru	140	26	17	1	0	184
	Kajiado	81	21	5	0	0	107
Intervention	Kakamega	111	48	12	1	0	172
	Lamu	29	3	3	0	0	35
	Homabay	120	48	16	0	0	184
	Nyandarua	54	24	2	0	0	80
	Total by Level	1058	272	96	4	0	1430

**Table 2:** Proposed Study Participants

Participant Category	Subcategory	Number of Participants
National Level Participants	National Treasury	2
	Ministry of Health	2
	Development Partners	2
	Controller of budget	1
	Auditor General	1
	Council of Governors	1
	Civil Society	2
County-Level Participants (per county)	County attorneys	1 per county (6)
	County Treasury	1 per county (6)
	County Department of Health	2 per county (12)
	Sub County Managers	2 per county (12)
	Health Facility Managers	5 per county (30)
	County Assembly	1 per county (6)
**Total Participants**		**83**

## Key Messages:

- **What is already known on this topic** – Existing evidence from high-income countries suggests that hospital autonomy has positive effects on service delivery.
- **What this study adds** – This study contributes by examining health facility autonomy at the primary healthcare level in a lower-middle-income country with semi-autonomous sub-national governments.
- **How this study might affect research, practice or policy** – Upon completion, this study will enhance our understanding of empowering frontline facility managers to make informed decisions, potentially influencing future research, healthcare practices, and patient experience.
