# Evaluating the effects, implementation experience and political economy of primary healthcare facility autonomy reforms within counties in Kenya: a mixed methods study protocol

**DOI:** 10.1136/bmjph-2024-001156

**Published:** 2024-10-16

**Authors:** Anita Musiega, Beatrice Amboko, Beryl Maritim, Jacinta Nzinga, Benjamin Tsofa, Peter Mwangi Mugo, Ethan Wong, Caitlin Mazzilli, Wangari Ng'ang'a, Brittany L Hagedorn, Gillian Turner, Anne Musuva, Felix Murira, Nirmala Ravishankar, Edwine Barasa

**Affiliations:** 1 Health Economics Research Unit, KEMRI-Wellcome Trust Research Programme, Nairobi, Kenya; 2 Health Systems and Research Ethics, KEMRI-Wellcome Trust Research Programme, Kilifi, Kenya; 3 Bill & Melinda Gates Foundation, Seattle, Washington, USA; 4 ThinkWell, Washington, DC, USA; 5 ThinkWell, Nairobi, Kenya; 6 Health Economics Research Unit, KEMRI-Wellcome Trust Research Programme, Kilifi, Kenya; 7 Nuffield Department of Medicine, Center for Tropical Medicine and Global Health, University of Oxford, Oxford, UK

**Keywords:** Economics, Community Health, Public Health

## Abstract

**Introduction:**

There is a growing emphasis on improving primary healthcare (PHC) services and granting frontline service providers more decision-making autonomy. In October 2023, Kenya enacted legislation mandating nationwide facility autonomy. There is limited understanding of the effects of health facility autonomy on PHC facilities performance. It is recognised that stakeholder interests influence reforms, and gender plays a critical role in access to health and its outcomes. This protocol outlines the methods for a study that plans to evaluate the effects, implementation experience, political economy and gendered effects of health facility autonomy reforms in Kenya.

**Methods and analysis:**

The research will use a before-and-after quasi-experimental study design to measure the effects of the reform on service readiness and service utilisation and a cross-sectional qualitative study to explore the implementation experience, political economy and gendered effects of these reforms. Data to measure the effects of autonomy will be collected from a sample of 80 health facilities and 1600 clients per study arm. Qualitative interviews will involve approximately 83 facility managers and policymakers at the county level, distributed across intervening (36) and planning to intervene (36) counties. Additionally, 11 interviews will be conducted at the national level with representatives from the Ministry of Health, the National Treasury, the Controller of Budget, the Council of Governors, the Auditor General and development partners. Given the uncertainty surrounding the implementation of the reforms, this study proposes two secondary designs in the event our primary design is not feasible—a cross-sectional study and a quasi-experimental interrupted time series design. The study will use a difference-in-difference analysis for the quantitative component to evaluate the effects of the reforms, while using thematic analysis for the qualitative component to evaluate the political economy and the implementation experience of the reforms.

**Ethics and dissemination:**

This study was approved by the Kenya Medical Research Institute Scientific and Ethics Review Unit (KEMRI/SERU/CGMR-C/294/4708) and the National Commission for Science, Technology and Innovation (NACOSTI/P/23/28111). We plan to disseminate the findings through publications, policy briefs and dissemination workshops.

**WHAT IS ALREADY KNOWN ON THIS TOPIC**

- Existing evidence from high-income countries suggests that hospital autonomy has positive effects on service delivery.

**WHAT THIS STUDY ADDS**

- This study contributes by examining health facility autonomy at the primary healthcare level in a lower-middle-income country with semiautonomous subnational governments.

**HOW THIS STUDY MIGHT AFFECT RESEARCH, PRACTICE OR POLICY**

- On completion, this study will enhance our understanding of empowering frontline facility managers to make informed decisions, potentially influencing future research, healthcare practices and patient experience.

## Background

 Primary healthcare (PHC) is an entire society’s approach to delivering timely, comprehensive and people-centred health services as close as possible to people’s everyday surroundings.^1^ PHC prioritises the delivery of essential health promotion and disease prevention, in addition to treatment, rehabilitation and palliative care, and is a crucial element in universal health coverage as it ensures equitable and efficient access to healthcare that prioritises the needs of individuals.^2^ First referral hospitals and peripheral outpatient health facilities are integral in delivering facility-based PHC services in low-income and middle-income countries (LMICs). Evidence shows that how health facilities are organised and managed determines their effectiveness and the extent to which they meet service delivery goals.^1 3^

Health facility autonomy is a crucial aspect of the organisational structure and management of health facilities. Health facility autonomy refers to the level of control and influence that health facilities have over key functions health facility administration, financial management, procurement, human resource management and strategic management.^4^ Historically, health facility autonomy reforms emerged as part of new public management prescriptions for hospital reforms and entailed both decentralisation of functions to hospitals and exposure to the market.^5^ The level of autonomy granted to public facilities has been shown to directly affect their performance.^2^ Unfortunately, in LMICs, most public PHC facilities lack autonomy over most of the managerial functions. For instance, less than 40% of facilities in LMICs have autonomy to retain and spend their revenue.^2^

Historically, health facility autonomy reforms have been introduced to enhance the efficiency of health facility operations and reduce their reliance on government budgets. Autonomy reforms have also been part of broader decentralisation reforms aimed at improved efficiency and equity of health systems as well as enhance local participation and accountability.^46^,^8^

However, like broader decentralisation reforms, there is mixed evidence regarding whether granting PHC facilities autonomy improves health facility performance in LMICs. For example, a study conducted in India found no significant relationship between health facility decision-making autonomy and performance.^9^ In Uganda, a study that explored the health facility decision space found increased autonomy to be positively associated with the availability of drugs but negatively associated with quality of care and performance management.^10^ In Tanzania, increased autonomy has had varying levels of effects on performance, linked to the functionality of the facility governance committees.^11^ The mixed evidence may be attributed to context-specific factors influencing the effects on autonomy.^12^ These mixed findings notwithstanding, there is increasing attention to leveraging health facility autonomy to improve the performance of health facilities and to enhance the role of the people in health service delivery, more so in PHC facilities. In addition, often health system reforms are experienced differently across genders, it is important that policy reforms consider the gendered impact to mitigate for unintended consequences.

Methodologically, most studies that have documented the effects of autonomy reforms have been qualitative^4 7 8 13^ and the few quantitative studies in this area are cross-sectional, presenting attribution challenges.^9^ This study proposes to adopt a mixed methods approach, incorporating a qualitative inquiry, and a range of quantitative methods (quasi-experimental before and after, cross-sectional and interrupted time series (ITS) designs) aimed at estimating the effect of autonomy reforms. The study also proposes to conduct a political economy and gender analysis of autonomy reforms, both of which have not been examined in previous studies of autonomy.

### Autonomy reforms in Kenya

Kenya has been on a path to improve service delivery through devolution and more recently increased investments in PHC services.^14^ Kenya devolved health service delivery to county governments in 2013. Health facilities enjoyed some level of autonomy before devolution (health facility administration, financial management, procurement, human resource management and strategic management), through the health management services fund for hospitals and health sector services fund for health centres and dispensaries, which was shown to be effective in enhancing service delivery.^13^ However, following the devolution of health services in 2013, health service delivery was reorganised with county health management teams (CHMTs) taking up the responsibility for the management of health services and usurping the autonomy of subcounty and facility management committees.^7^

Over time, various counties have acknowledged the need for facility managerial autonomy and have allowed facilities some level of financial and procurement autonomy.^15^ Across these counties, this has been done through various means including enactment of county-level legislation and/or adoption of county-level policies to facilitate health facility autonomy. However, many counties have continued to limit the autonomy of health facilities. At the time of writing this protocol, the country had recently enacted a new Facility Improvement Funds (FIF) law that aimed at according some level of financial and procurement autonomy to public sector health facilities across the country.^16^ The law requires that facilities in the country are allowed to retain and spend their own source revenue. This law mandates all counties to implement autonomy reforms. However, the government was yet to establish guidelines for the implementation of this new law. Furthermore, due to the devolution of the management of health services in the country, the adoption and implementation of this new law is likely to depend on individual county leadership, unlike before the devolution when national laws were adopted exactly as guided by the national government. This will likely lead to variations in uptake of facility autonomy across counties in terms of both time of implementation and mechanism of implementation. These variations in implementation provides an opportunity to evaluate the effectiveness and implementation experience of autonomy reforms.

In addition, it is crucial to consider the gendered effect of these autonomy reforms. Within Kenya, and in other African countries, there is a gendered influence in health leadership progression, with women often lagging.^17 18^ In Kenya, it has also been shown that policies that are gender sensitive have had a positive influence on women in leadership.^17^ It is important to assess whether the autonomy reforms will reflect the representation of women in decision-making. In addition, gender dynamics often play a significant role in healthcare utilisation, access and outcomes.^19 20^ This study will explore whether the autonomy reforms have differential effects on men and women, considering factors such as health system leadership positions, access to services, health outcomes and the involvement of women in decision-making processes at the community and facility levels. Addressing gender-specific challenges and opportunities within the framework of PHC and facility autonomy will contribute to a more comprehensive and inclusive understanding of the healthcare system in Kenya.

It is important to document these autonomy reforms, their successes and failures, to provide learning points for counties willing to implement similar reforms in the future and for other LMICs keen on enhancing PHC provider autonomy. This study seeks to examine the effects, implementation experience, political economy and gendered effect of facility autonomy reforms among counties in Kenya.

## Methods

To address the above objectives, this study will adopt a concurrent mixed-method study design. The quantitative component of the study will entail a before and after quasi-experimental study as the primary design. The qualitative study will entail (1) a process evaluation and (2) a political economy analysis (PEA) of the facility autonomy reforms. Both the quantitative and qualitative elements of the study will incorporate a gender lens. This study started in January 2023 and will end in February 2026.

### Evaluation framework: theory of change (ToC)

**Figure 1:**
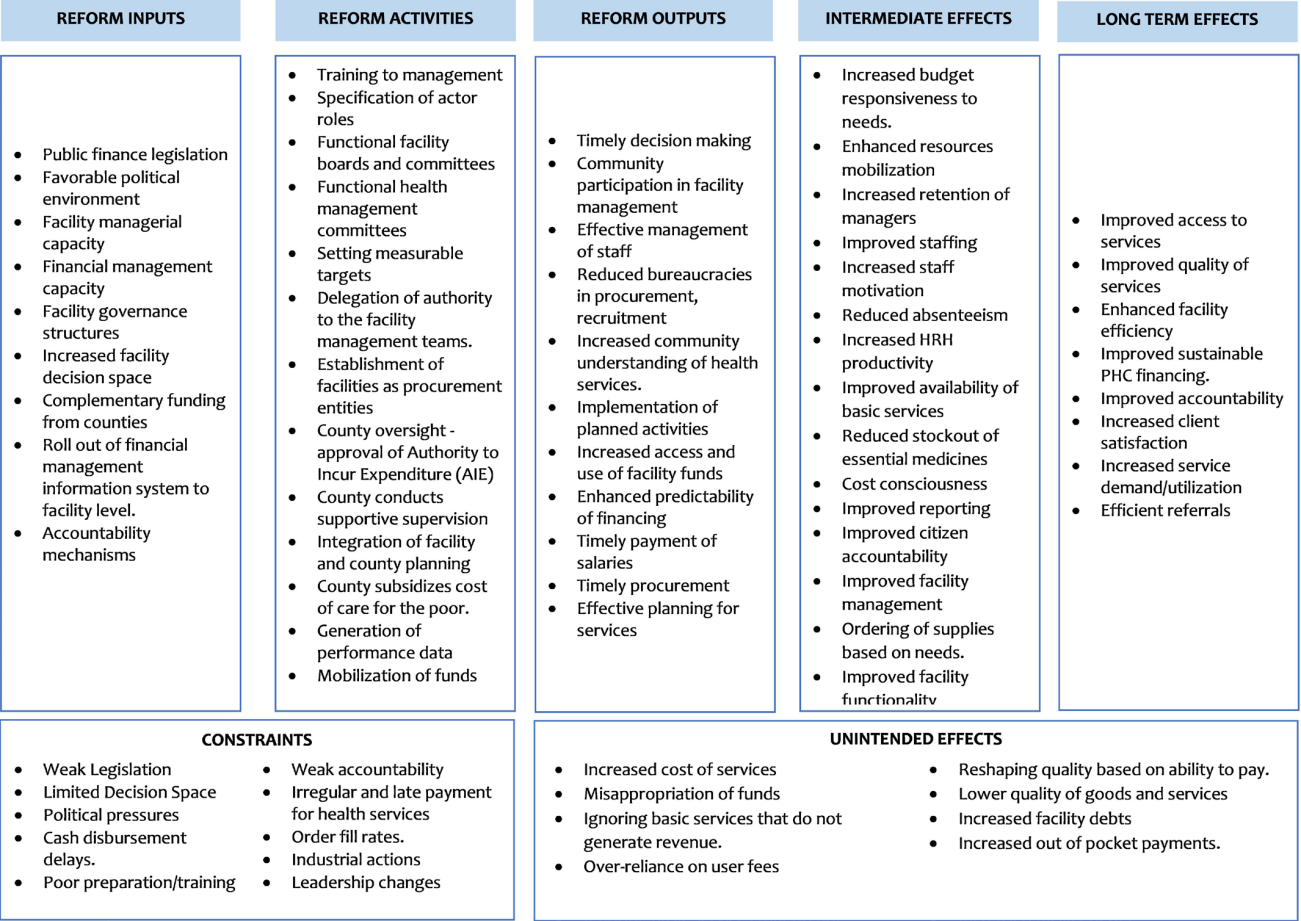
Theory of change. PHC, primary healthcare.

This study will be guided by a ToC (figure 1) which we developed from reviewing literature on the relationship between facility autonomy and facility performance and a co-creation workshop that brought together 25 health sector stakeholders drawn from the national and county level and government and development partners. The ToC assumes that facility autonomy spans key functions of the facility including strategic management, financial, procurement, human resource management and facility administration.^7^ This autonomy will require effective legislation, a favourable political environment, facility managerial capacity, facility capacity to claim reimbursements, facility governance structures, increased facility decision space, complementary funding from counties, roll-out of financial management information system to facility level and established accountability mechanisms.^4^ The key activities will include training to management, specification of actor roles, functional facility boards and committees, functional health management committees, setting measurable targets, delegation of authority to the facility management teams, establishment of facilities as procurement entities, county oversight - approval of Authority to Incur Expenditure, supportive supervision by the county and sub-CHMTs, integration of facility and county planning, subsidisation of the cost of care for the poor to guard against inequity, generation of performance data and mobilisation of funds.^47 21^,^23^

The immediate outputs expected from the inputs and activities include timely decision-making, enhanced community participation in facility,^7 24 25^ effective management of staff,^24 25^ reduced bureaucracies in procurement and recruitment, increased community awareness of services implementation of planned activities,^7 26^ increased access and use of facility funds,^7 26^ enhanced predictability on financing,^4^ timely payment of salaries,^4 26^ timely procurement^4 26^ and, finally, effective planning for services.

The expected medium-term outcomes include increased budget responsiveness to needs, enhanced resource mobilisation, increased retention of managers, improved staffing, increased staff motivation,^7 26 27^ reduced absenteeism, increased Human Resources for Health (HRH) productivity,^4 24 25^ improved availability of basic services, reduced stockout of essential medicines, cost consciousness, improved reporting, improved citizen accountability and participation, improved facility management and ordering of medicines and supplies based on need. These medium-term goals should then result in the achievement of the following long-term goals: improved access to services, improved quality of services, enhanced facility efficiency, improved sustainable PHC financing, improved accountability, increased client satisfaction, increased service demand/utilisation and efficient referrals.^4 7 26 27^

From the literature, the effect of autonomy reforms is subject to context which is determined by institutional, socioeconomic and geographic factors.^12^ As such, we will monitor constraints that are likely to influence the effectiveness of the reform including weak legislation, dysfunctional boards/committees, limited decision space, political pressures, cash disbursement delays and poor preparation/training.^4 8^

Finally, autonomy reforms may have unintended negative consequences including increased cost of services, misappropriation of funds, ignoring basic services that do not generate revenue, over-reliance on user fees, reshaping quality based on ability to pay, lower quality of goods and services, increased facility debts and increased out of pocket payments. We will monitor the possible unintended consequences and document the efforts by counties to mitigate them.

### Evaluating the effects of autonomy reforms

#### Primary study design

We will employ a before and after quasi-experimental study to measure the effects of health facility autonomy on health facility readiness, functionality, service utilisation and client satisfaction of health facility autonomy reforms. We will conduct an assessment before implementation of the FIF reforms and an assessment after 12 months of implementation of the FIF reforms. The primary outcome of interest will be health facility readiness.

#### Secondary study design

There are uncertainties about the roll-out of autonomy, with possibilities that might compromise the proposed study design (quasi-experimental before and after design). The pace of autonomy reforms roll-out could be slow, with the implication that the selected intervention counties do not convert to intervention counties (ie, autonomy reforms are not implemented in these counties over the 12-month study period). To mitigate this, we will sample four additional intervention counties that have already implemented autonomy reforms. We will use a cross-sectional study design comparing these four counties that are already implementing autonomy, with the counties that do not have autonomy (control counties). This design will also ensure that we can measure effects in the event the time of the study is not sufficient to measure effects within counties that are just rolling out autonomy.

Further, we will use an ITS design to evaluate the effects of the reforms in the counties that will have rolled out the reforms across the country. We will collect and analyse monthly District Health Information System (DHIS) data on key performance indicators from PHC facilities across selected counties in Kenya, spanning 24 months before the introduction of autonomy reforms in a county and 24 months after its introduction. Exact times for pre-intervention and post-intervention phases will vary based on the exact time that autonomy reforms were introduced in a county.

#### Study population

We intend to assess selected counties, selected facilities and clients receiving services from the selected facilities. For the county assessment, the study population will be the 47 counties from which we have selected 12 counties—4 implementing autonomy reforms, 4 planning to implement and 4 controls. For the facility assessment, the study population will be all county-owned public facilities within the selected counties across all level 2-6 of service provision level 2 (dispensaries), level 3 (health centers) level 4, 5 and 6 (hospitals) (table 1). Level 1 in Kenya comprises community health services, these will be excluded as at the time of the protocol, there were minimal to no mechanisms for payment for services provided at that level. For the client exit interviews, the study population will be all patients or carers whose patients had received services from the selected facilities on the day and time of the survey. These will include patients or carers seeking services such as outpatient services, antenatal services, postnatal services, child welfare services, management of chronic illness and maternity services and those discharged from inpatient care.

**Table 1:** Selected counties with distribution of facilities

Group	County	Level 2	Level 3	Level 4	Level 5	Level 6	Total facilities in county
Controls	Tana River	44	5	3	0	0	52
	Migori	115	19	14	0	0	148
	Embu	88	9	4	1	0	102
	Kirinyaga	47	22	4	0	0	73
Already implementing	Makueni	189	39	12	0	0	240
	Mombasa	40	8	4	1	0	53
	Nakuru	140	26	17	1	0	184
	Kajiado	81	21	5	0	0	107
Intervention	Kakamega	111	48	12	1	0	172
	Lamu	29	3	3	0	0	35
	Homabay	120	48	16	0	0	184
	Nyandarua	54	24	2	0	0	80
	Total by level	1058	272	96	4	0	1430

#### Sample size and sampling

There will be three levels of sampling for this study. The first level is county sampling, where we will purposively sample four counties already implementing autonomy reforms, four planning to implement the intervention and four not implementing as control. From the sampled counties, we will randomly sample 80 health facilities per group, assuming a mean difference of 10% in facility readiness between the two groups and a population variance of 400 across the groups using 80% power, 95% confidence level and a design effect of 1.2. Because the autonomy reforms have the biggest effects on facilities that raise revenue, we will sample all facilities from levels 4–6 from the selected counties. Leve 4-6 facilities are allowed by law to charge user fees. For the remaining facilities, we will then randomly sample levels 2 and 3. The numbers will be proportionate, targeting a maximum 20 facilities per county. For clients, we will randomly sample a minimum of 1600 clients exiting the health facilities per group, assuming a mean difference of 10% in client satisfaction between the two groups and a population variance of 55 across the groups using 80% power, 95% confidence level and a design effect of 3. To calculate the variance, we took an average of the outcome of the interest in the control and intervention (expected).^28^,^30^ We divide the 1600 clients equally among the selected facilities to achieve a minimum sample of 20 per facility as is recommended for cluster randomised trials.^28 30^ The following sample size calculation formula was used:

,$$n=\left(Z_{\alpha /2}+Z_{\beta \;}\right)sqrd\times 2\times \;\sigma^{2}\;/d^{2}$$

where Z_α/2_=1.96 (5% significance), Z_β_=0.84 (80% power), σ^2^=population variance of 59 (SD of 19 per group) and d=mean difference of 8% between the two groups.

#### Data collection

We will use trained research assistants to collect data using structured questionnaires on a web-based electronic platform. These questionnaires will be administered in the period preceding and 12 months after the roll-out of autonomy reforms. The health facility questionnaire will be administered to the health facility in charge and other health workers with information on the various sections. It will collect data on the facility profile, target population, services provided, availability of medicine, availability of laboratory services, availability of basic equipment and service utilisation (online supplemental table 1). The health facility assessment questionnaire will evaluate the impact of autonomy on service availability, access to basic equipment, availability of medicines, service utilisation and facility financing (see online supplemental table 1). The client exit interview tool will measure the effects of health facility autonomy on some subjective and objective indicators around service availability and client satisfaction. The exit interviews will seek to establish whether health facility autonomy influences the availability of services and client satisfaction. It will entail questions about the services sought, whether the clients received the services, the client’s perspective on the cost of services, the client’s perspective on facility cleanliness and health worker attitude, how often the clients visit the health facility and whether clients are satisfied with the services (online supplemental table 1).

#### Data analysis

We will compute means, medians and SD of all study variables and key sample characteristics at the baseline and end line. To determine the effects of autonomy reforms on service utilisation and client satisfaction, we will use the quasi-experimental difference-in-difference (DiD) analysis. We will first compute the differences in means between the baseline and end line for each study group, then the differences in mean between the intervention and control.

Thereafter, we will estimate the effect using multivariate regression analysis. This means that we will calculate the DiD while controlling for other covariates that are likely to affect outcomes such as facility average insurance coverage, average distance to facility for facility catchment population, health worker density and facility size. The unit of analysis will be the health facility, and the outcomes of interest will be service utilisation and client satisfaction.

### Qualitative study

#### Study design

The cross sectional qualitative study will address objectives 2, 3 and 4 of the study. We will employ a cross-sectional qualitative study design to (1) assess the gendered effect of facility autonomy reforms, (2) assess fidelity and implementation experience of hospital autonomy reforms and (3) examine the political and economic factors that influence hospital autonomy reforms.

##### Gendered analysis

The gender analysis will entail examining the reform for the gendered effects. We propose to use the 4R method to conduct the gendered analysis—responsibility, in terms of the gendered allocation of responsibility, the gendered allocation of resources, the reason behind the gendered allocations and the realisation—what would improved gender equality look like.^31^

##### Process evaluation

The process evaluation of autonomy reforms will entail examining the emergence of autonomy reforms, the fidelity of the implementation reforms, the implementation processes and the reasons for the resultant outputs or outcomes. In examining the emergence of the autonomy reforms, we examine the factors that led to the reforms and the adopted approaches. In examining the implementation fidelity, we will seek to compare the reforms as outlined in the FIF guidelines and laws (de jure policies) and what is implemented ‘on the ground’ (de facto policies). In examining the processes, we will seek to document actors’ experiences, the implementation process, adaptations triggered by the reforms, unintended consequences and how contextual issues shaped the reforms. In examining the resultant outputs or outcomes, we will present the results of the quantitative aspects of the study and seek to enrich the findings with qualitative data. We will examine implementation fidelity based on what is articulated in the implementation plans of health facility autonomy reforms in the intervention counties.

##### Political economy analysis

We will conduct a problem-driven PEA. This will entail the exploration of actor interests and incentives and the de jure and de facto policies and how they interact to influence health facility autonomy.^32^ We propose to use a modified World Bank problem-driven PEA.^33^ We adopt this approach because, from the literature, the success of autonomy reforms is linked to the institutions, actors, historical conditions and power dynamics.^4 7 15 26^ The problem-driven PEA has three aspects: (1) description of the problem, (2) institutional analysis which entails mapping out all the government organisations involved in (financial) autonomy and the legal and policy documents that relate to the problem and, finally, (3) identify political economy drivers—specifically this will entail describing why things exist the way they do.^34^ In exploring the drivers, we will consider three structures of drivers—structural (historical legacies, status of poverty, rent/rent distributions), institutional and actors/stakeholders.^34^ To assess the stakeholders, we propose to adopt Sparkes *et al*’s classification of actors: interest group politics, bureaucratic politics, budget politics, leadership politics, beneficiary politics and external politics.^32^

### Study population

The study population will include both national-level and county-level stakeholders involved in health facility autonomy reforms. We will include national, county and facility stakeholders involved in health financing and PHC service delivery decision-making. These will include stakeholders from health departments, finance departments and county assembly, who were involved in the health financing reforms.

### Sample size and sampling

The main data collection method will be in-depth interviews and document reviews. For the in-depth interview, we anticipate interviewing about 83 participants; however, this will be guided by the need to have county balance as the reforms are county specific and saturation of data. These respondents will be purposively selected based on their knowledge and involvement in health facility autonomy reforms. Based on the number of people holding these positions, we anticipate conducting a total of 11 interviews at the national level and 12 interviews each from 3 already intervening counties and 3 planning to intervene counties (table 2). For the document reviews, we will review documents related to health facility autonomy including the PFM laws, budget documents, facility reports, minutes and county bills.

**Table 2:** Proposed study participants

Participant category	Subcategory	Participants (n)
National level participants	National Treasury	2
	Ministry of Health	2
	Development partners	2
	Controller of Budget	1
	Auditor General	1
	Council of Governors	1
	Civil society	2
County-level participants (per county)	County attorneys	1 per county (6)
	County treasury	1 per county (6)
	County department of health	2 per county (12)
	Subcounty managers	2 per county (12)
	Health facility managers	5 per county (30)
	County assembly	1 per county (6)
Total participants		83

### Data collection

We will collect data using in-depth interviews and document reviews. In-depth interviews will target national-level and county-level officials involved in facility autonomy reforms (table 2). We will collect relevant data from documents that relate to facility autonomy reforms.

### Qualitative data analysis

The audio recordings will be converted into text format using Microsoft Word and then processed in NVIVO software for coding. Analysis of the data will follow a thematic approach. Thematic analysis is a method that guides the identification, organisation, description, analysis and reporting of themes founds in a data set.^35^ The data analysis will follow six steps: familiarising with the data, generating initial codes, searching for themes, reviewing themes, naming themes and writing the report.^35 36^ We will report the gendered effects of the reforms, implementation process and political economy as detailed in the list of analysis areas and outcomes.

### Data management

#### Methodological rigour

To ensure the robustness of our research, we will implement various measures. First, we will conduct a literature review and a co-creation workshop to validate the ToC that will guide the development of indicators. Second, we will document our entire research process and provide clear rationales for our study choices. Third, we will align our analysis with the ToC, using both quantitative and qualitative designs for triangulation. Fourth, we will extensively train our data collectors and conduct pilot studies in Kisumu, Mombasa, Makueni and Kiambu counties. Fifth, when developing tools, we will adopt measures from established instruments, control for extraneous variables use a large randomly selected sample. Methodological rigour in qualitative aspects will involve peer debriefing, reflexivity and respondent validation during a dissemination workshop, while a pilot study will test and refine our research protocols before the main study. Adjustments based on pilot feedback will contribute to our overall methodological robustness in future research endeavours.

We will ensure data safety; no identifiable information will be stored with survey responses. Survey responses will be maintained on secure, password-protected servers at Kenya Medical Research Institute (KEMRI)-Wellcome Trust Research Programme (KWTRP). Deidentified data files will be shared with the research team via secure file transfer and will be maintained on password-protected devices.

We will develop a database capturing data from the questionnaires in REDCap or equivalent software in collaboration with the KEMRI-Wellcome Nairobi data team. We will check the data for errors and import it into R for analysis. We will collate data from these questionnaires on a central server maintained by the KWTRP after being deidentified and encrypted for transmission over the internet. Deidentified data shared with KWTRP will be stored in secure servers with password-protected access and as guided by KEMRI-Wellcome Trust ICT and Data Management policies. Data held on these servers are backed up in similar password-protected mirror servers at another location within the KEMRI-Wellcome Trust.

### Ethical considerations

This study has received ethics approval from the Scientific and Ethics Review Unit (SERU) at KEMRI, approval number KEMRI/SERU/CGMR-C/294/4708. The study has also received approval from the National Commission for Science, Technology and Innovation (NACOSTI), approval number NACOSTI/P/23/28111. Recognising potential participant anxiety related to autonomy within counties, we will conduct low-risk interviews in private locations to ensure confidentiality. All study data, including transcripts and documents, will be deidentified and securely stored. Informed consent will be provided to all participants, with participants receiving comprehensive information sheets and consent forms in either English or Swahili based on their preferences.

### Communication and dissemination of results

Throughout the study period, we plan to conduct dissemination workshops with policymakers. Initially, we organised a co-creation workshop during the protocol development phase to validate the ToC. We will host a dissemination workshop following the preliminary analysis of baseline data and another after the final data collection and analysis. Our study is integrated within the Ministry of Health and the Council of Governors, who are key collaborators. Additionally, we intend to publish various aspects of the study and present our findings at conferences.

## Supplementary material

10.1136/bmjph-2024-001156 online supplemental file 1

## Data Availability

Data sharing not applicable as no datasets were generated and/or analysed for this study. All data relevant to the study are included in the article or uploaded as supplementary information.
