# Supplementary material for: Evaluating the effects, implementation experience and political economy of primary healthcare facility autonomy reforms within counties in Kenya: a mixed methods study protocol
Source: BMJ Public Health. 2024 Oct 16;2(2):e001156. doi: 10.1136/bmjph-2024-001156 (PMC11737602; doi:10.1136/bmjph-2024-001156)
Supplement: online supplemental file 1 [file bmjph-2-2-s001.pdf]

Table 2: Overview of Core Indicators for each of the surveys

**Type of effects**	**Expected output**	**Indicator**	**Data Source**
Health facility readiness and functionality	Improved availability of basic equipment	% availability of basic equipment	Facility assessment
	Reduced stockout of essential medicines	% availability of essential medicines	Facility assessment
	Low order fill rates - constraints	Order fill rates	Facility/county/KEMSA reports
	Enhanced availability of services	% availability of basic diagnostic tests	Facility assessment
	Enhanced availability of services	% patients sent out to conduct diagnostic procedures	Client Exit Interview
	Enhanced availability of services	% patients sent to purchase medication outside of the facility	Client Exit Interview
	Improved access to services	% Services offered by the facility	Facility Assessment
	Improved Facility Functionality	% facilities with power outage in the last 7days	Facility Assessment
	Improved Facility Functionality	% facilities with insufficient water supply at any time in the past year	Facility assessment
	Improved Facility Functionality	% Facilities with functional communication equipment	Facility Assessment
Service Utilization	Increased service utilization	Number of outpatient visits	DHIS

	Increased service utilization	Number of inpatient admissions	DHIS
	Improved access to services	Number of health facility deliveries	DHIS
	Increased service utilization<br>Improved access to services	Number of ANC visits	DHIS
	Increased service utilization<br>Improved access to services	Number of children immunized	DHIS
	Increased service utilization	Number of PNC visits	DHIS
	Increased client satisfaction	% patients willing to visit the facility again	Client Exit Interview
	Increased client satisfaction	% patients willing to refer their friends to the facility	Client Exit Interview
	Improved access to services<br>Reduced unnecessary referrals	% clients receiving required services	Client Exit Interview
Accountability	Improved accountability<br>Effective planning and budgeting	% facilities whose management committee met in the last month	Facility Assessment
	Community participation in facility management<br>Improved accountability	% Facilities with committees with community representative	Facility Assessment

	Improved reporting	% Facilities reporting monthly supervision by sub-county	Facility Assessment
	Improved accountability	%facilities with functional health management committees	Facility assessment
Facility Financing	Enhanced Resource Mobilization	Total revenue raised by Facility in Financial Year	Facility Assessment
		Total NHIF Claims made	Facility Assessment
		Total NHIF Claims paid	Facility Assessment
	Over-reliance on user fees	Total revenue received by the facility from out of pocket payments	
	Improved sustainable financing to PHC	% Funds absorbed facility in the Financial Year	Facility Assessment
	Increased budget responsiveness to needs	Difference between AWP budgets and PBB budgets	Facility Assessment
Human Resources for Health	Improved staffing	Number of staff in the facility	Facility Assessment
	Improved staffing	Facility staffing as a % of staffing norms	Facility Assessment

	Reduced absenteeism	% workers on duty who are available at the time of the visit	Facility Assessment
	Increased HRH productivity	Caseload per provider per day	Facility assessment
Quality of care	Increased client satisfaction	% reporting satisfaction with the quality of care	
	Increased client satisfaction	Average waiting time at the facility	Client Exit Interview
Data quality	Improved reporting	% Facilities reporting on DHIS	DHIS
	Improved reporting	% Facility with Functional IT equipment	Facility Questionnaire
Gendered effects of autonomy reforms	Gender balance in leadership positions	% women in Facility leadership positions	Facility Questionnaire
	Equity in access to health services	% men accessing health services in facilities	Facility questionnaire
